# A New Crystal Form of the SARS-CoV-2 Receptor Binding Domain: CR3022 Complex—An Ideal Target for In-Crystal Fragment Screening of the ACE2 Binding Site Surface

**DOI:** 10.3389/fphar.2020.615211

**Published:** 2020-12-14

**Authors:** Charlie Nichols, Joseph Ng, Annika Keshu, Franca Fraternali, Gian F. De Nicola

**Affiliations:** ^1^ British Heart Foundation Centre of Excellence, Department of Cardiology, Rayne Institute, St Thomas’ Hospital, King’s College London, London, United Kingdom; ^2^ The Randall Centre for Cell and Molecular Biophysics, New Hunt’s House, King’s College London, London, United Kingdom

**Keywords:** in-crystal fragment screening, CR3022, drug discovery, PPI inhibitors, SARC-CoV-2-RBD

## Abstract

In-crystal fragment screening is a powerful tool to chemically probe the surfaces used by proteins to interact, and identify the chemical space worth exploring to design protein-protein inhibitors. A crucial prerequisite is the identification of a crystal form where the target area is exposed and accessible to be probed by fragments. Here we report a crystal form of the SARS-CoV-2 Receptor Binding Domain in complex with the CR3022 antibody where the ACE2 binding site on the Receptor Binding Domain is exposed and accessible. This crystal form of the complex is a valuable tool to develop antiviral molecules that could act by blocking the virus entry in cells.

## Introduction

**FIGURE 1:**
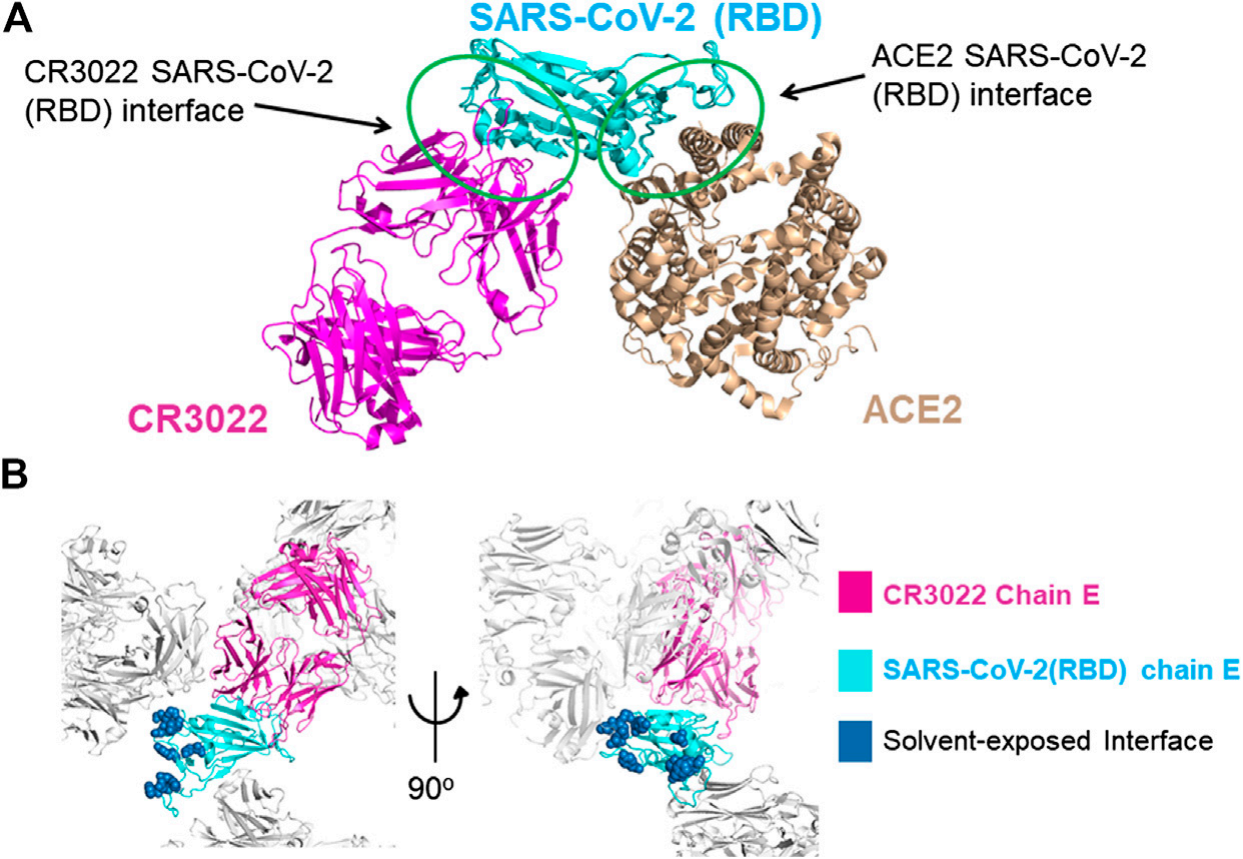
**(A)** Superimposition of the X-ray structures of the SARS-CoV-2-RBD CR3022 (PDB 6W41) and SARS-CoV-2-RBD:ACE2 (PDB 6MOJ) complexes. The antibody and the human receptor use two distinct non-overlapping surfaces to interact with the virus receptor binding domain. **(B)** zoom-in on the lattice reconstruction of the SARS-CoV-2-RBD:CR3022 complex from the PDB 6ZLR. The Chain E of the complex in the ASU is colored, the ACE2 binding site on the RBD is shown in dark blue, it highlights that the entire binding site is solvent accessible on chain E.

A new form of viral pneumonia caused by the coronavirus named SARS-CoV-2 was first reported in Wuhan China at the end of 2019 and has since been declared a pandemic by the World Health Organization. It is believed that the virus targets the ACE2 receptor on the surface of human cells (Walls et al., 2020). The X-ray structure of the complex between the Receptor Binding Domain (RBD) of the viral spike glycoprotein SARS-CoV-2 and the ACE2 human receptor has been solved and the surface used by the two proteins to interact identified (Lan et al., 2020). To date there is no antiviral drug that targets the SARS-CoV-2-RBD. An antiviral molecule that binds to the RDB of the SARS-CoV-2 spike protein and inhibits its interaction with the human ACE2 receptor could become a valuable tool in the fight against the virus. We have previously shown that X-ray crystallography combined with fragment screening can be used to chemically probe the surfaces used by proteins to interact, and the outcome of such screens can be used to design protein-protein inhibitors (Nichols et al., 2020). In here we describe a new crystal form of the SARS-CoV-2 Receptor Binding Domain (RBD) in complex with the CR3022 antibody where the ACE2 binding site on the RBD is exposed and accessible. An in-crystal fragment screen using this crystal form would allow the identification of the chemical space worth exploring to design antiviral molecules against the ACE2 binding site on the SARS-CoV-2 Receptor Binding Domain (RBD). To date three structures of the SARS-CoV-2 RBD in complex with the antibody CR3022 (PDB: 6YM0, 6YLA, 6W41) have been submitted to the PDB, the analysis of the structure of the complex shows that the CR3022 antibody does not compete with the ACE2 binding site on the surface of the RBD (Figure 1A); among the submitted pdbs 6YLA and 6W41 have higher resolution, 2.4 Å and 3.1 Å respectively, but only part of the ACE2 binding site on the RBD is free from crystal lattice contacts therefore not suitable for in-crystal fragment screening whereas in 6YM0 the target area is more exposed but with a resolution of 4.4 Å the system is also not suitable for in-crystal fragment screening. In the crystal form we present here (PDB 6ZLR), the resolution of the complex is 3.1 (Table 1) in chain E the entire ACE2 binding site on the RBD is free from lattice contacts and accessible to solvent, making such form an ideal target for chemical exploration with fragments of that surface (Yuan et al., 2020; Huo et al., 2020).

**TABLE 1:** Values in parentheses are for the highest resolution bin.

Data collection		
Beamline	I24, diamond	
Detector	Pilatus 3 6 M	
Space group	*P*4_1_2_1_2	
Unit-cell parameters		
*a* (Å)	207.13	
*b* (Å)	207.13	
*c* (Å)	199.87	
α (°)	90	
β (°)	90	
γ (°)	90	
Wavelength (Å)	0.999	
Resolution (Å)	68.028–3.09	
Total no. of reflections	338,143 (20,175)	
Unique reflections	72,060 (4,356)	
Multiplicity	4.7 (4.6)	
Completeness (%)	91.1 (90.6)	
R_*merge*_	0.226	
*R*_*pim*_	0.125 (0.569)	
〈*I*/σ(*I*)〉	4.6 (1.5)	
CC_1/2_	0.933 (0.415)	
Refinement		
Resolution (Å)	68.028–3.09	
No. of atoms	14,747	
*R*_*work*_ (%)	21.76	
*R*_free_ (%)	24.72	
R.m.s. deviations‡	—	
Bond length (Å)	0.008	
Bond angle (°)	1.55	
Mean *B* factor (Å^2^)	55.6	
Ramachandran plot	—	
Favored (%)	93.29	
Allowed (%)	5.28	
Outliers (%)	1.43	
PDB code	6ZLR	

## Methods

The SARS-CoV-2-RBD and CR3022 proteins were provided by Prof Ian Wilson, both in 150 mM NaCl and 20 mM Tris pH 7.5. The proteins were mixed in a 1:1 ratio to give 15 mg/ml complex, aged for 24 h at 4 °C and crystallized by the sitting-drop vapor diffusion method at 20 °C. Droplets were dispensed using a Mosquito robotic dispensing system with 400 nL of protein + 400 nL reservoir solution (0.1 M sodium malonate, 0.1 M Tris pH7.7, 21–30% PEG1000). Crystals were flash-frozen by immersion in liquid nitrogen and diffraction data were collected at the Diamond Synchrotron Facility, beamline I24. Data were processed and integrated with Dials, scaled with Aimless and solved by molecular replacement with Phaser (McCoy et al., 2007) using pdb models 6W41 and 6YLA. Both molecular replacement models were refined with Refmac (Pannu et al., 1998) using jelly-body refinement and global NCS restraints. The 6W41 derived model was then rebuilt in Coot (Emsley et al., 2010) using both map-sets as a guide and re-refined with Jelly-body refinement and local NCS restraints. The final coordinates and structure factors were deposited in the PDB, deposition ID: 6ZLR.

## Analysis

### SARS-CoV-2-RBD: CR3022 Complex: Lattice Comparison of the Available Crystal Forms (6W41, 6YLA and 6ZLR)

The first structure of the complex (PDB 6W41) was reported by prof. Wilson’s group, the analysis of the structure of the complex shows that the CR3022 antibody does not compete with the ACE2 binding site on the surface of the RBD (Figure 1A), (Yuan et al., 2020) soon after a second structures of the complex was reported PDB 6YLA (Huo et al., 2020).

The RBD constructs and the FAB-CR3022 heavy chain used to generate pdbs 6W41 and 6YLA are different in the N and C terminal regions of the RDB construct and there is a 2-residue deletion in the FAB-CR3022 heavy chain sequence. The two structures use different crystallization systems: 6W41 - sodium acetate pH 4.6/ammonium sulphate and glycerol, 6YLA—tris pH7.5/PEG1000 and sodium malonate, cryoprotection by addition of DMSO. The extended N-terminal region of the 6W41 RBD-construct makes it unlikely that it could crystallize in the 6YLA lattice, but we reasoned that the small molecule decoration might enhance the resolution of the 6W41 lattice. We therefore trialled grid-screens based on different combinations of components from both systems. From this we observed that the RBD:CR3022 complex used to generate pdb 6W41 will also crystallize in conditions similar to those used to generate pdb 6YLA. The crystals were thin rods with similar morphology to those observed in the 6W41 system, but subsequent synchrotron testing revealed that the thin rods were in fact a novel lattice different to both 6W41 and 6YLA. The 6W41 ASU contains one copy of the trimeric RBD:CR3022 complex, the RBD ACE2 binding site symmetry partners with itself forming a homo-dimeric crystal contact surface that occludes a substantial portion of the ACE2 binding site. Lattice 6YLA contains two copies of the complex in the ASU, one symmetry partners with itself generating the same RBD homo-dimeric contact as in lattice 6W41. The second forms a crystal contact with the light chain of FAB CR3022; the ACE2 binding surface on the RBD of this copy is more exposed than in 6W41, but still partially occluded by the crystal contacts. In pdb 6ZLR, the subject of this report, there are three copies of the complex in the ASU. Two copies symmetry partner with each other generating the same homo-dimeric lattice contact as in lattice 6W41 (chains A, D), but the third has the ACE2 binding site on the RBD projecting out into a 60 Å diameter solvent channel that runs all the way through the crystal lattice (chain E) (Figure 1B). As a result, the ACE2 binding site is completely solvent exposed, with 15–20 Å minimum separation from the nearest lattice contact.

## Conclusion

Since a new form of viral pneumonia caused by the coronavirus named SARS-CoV-2 was first reported in Wuhan China at the end of 2019 a considerable effort has been put in to discover an effective treatment against it. Some progress has been made with the management of patients hospitalized with Covid-2, and several clinical trials are ongoing to develop a vaccine and repurpose approved drugs, still to date there is only one approved antiviral drug, remdesivir, that directly targets the virus and it has be suggested to have a modest effect in reducing recovery time in hospitalized patients diagnosed with Covid-19 (Beigel et al., 2020; Agostini et al., 2018; Warren et al., 2016). The development of other antiviral molecules that directly target SARS-CoV-2 will be useful tools to fight the continuing spreading of the virus worldwide.

We have identified a suitable crystal form of the SARS-CoV-2-RBD:CR3022 complex to use to run an in-crystal fragment screen where the target is the ACE2 binding site on the RBD of the SARS-CoV-2 spike protein. In the crystal form we present here the accessible space surrounding the target area is such that there is room to accommodate and soak in expanded chemical elaborations of the initial hits allowing a genuine structure guided optimization. The outcome of these screens should be able to identify the chemical space worth exploring to design new antiviral molecules capable of interfering with the entrance of the virus into human cells.

## Data Availability Statement

The model and the map files are also available in the Supplementary Material. The datasets presented in this study can be found in online repositories. The names of the repositories and accession numbers can be found below: Protein data bank, 6ZLR.

## Author Contributions

All authors listed have made a substantial, direct, and intellectual contribution to the work and approved it for publication.

## Conflict of Interest

The authors declare that the research was conducted in the absence of any commercial or financial relationships that could be construed as a potential conflict of interest.
